# 

**Published:** 1881-12

**Authors:** 


### CONTENTS.

### ORIGINAL:

PAGE.

Volume One.—Editorial, . . . . . . . . . . . 561

What is Homœopathy? C. H. Lawton, M. D., Wilmington, Del., . . 562

Unproved Remedies and Dr. Berridge. P. P. Wells, M. D., Brooklyn, N. Y., . . . . . . . . . . . . 566

Aconitum Napellus in its relation to the Female Sexual System. C. Carleton Smith, M. D., Philadelphia, . . . . . 569

Diphtheria, Bacteria, and Dr. Wells. Rollin R. Gregg, M. D., Buffalo, N. Y., . . . . . . . . . . . . 571

Notes from our Scrap-Book. E. J. L., . . . . . . . 578

A New Discovery in the Service of Homœopathy. Translated from "*Populare Zeitschrift fur Homœopathy*," Jan. 1881, by Prof. Manuel Græter, II., . . . . . . . . . . . 580

Clinical Reflections. Ad. Lippe, M. D., Philadelphia, . . . . 587

### CLINICAL BUREAU:

Puerperal Mania—with History of a Case. John Macfarlan, M. D., Denver, Col., . . . . . . . . . . . 591

Cases from Practice. D. C. McLaren, M. D., Montreal, . . . 597

Genito-Urinary Therapeutics, . . . . . . . . . 699

PERISCOPE, . . . . . . . . . . . . . 600

### BOOK NOTICES, REVIEWS, ETC.

The Homœopathic Treatment of Diphtheria. By De Forest Hunt, M. D., . . . . . . . . . . . . 603

The Homœopathic Physician's Visiting List and Pocket Repertory. By Robert Faulkner, M. D., . . . . . . . . 603

The Physician's Quarterly Record and Statement. By Wm. Jefferson Guernsey, M. D., . . . . . . . . . . . 603

Pamphlets Received, . . . . . . . . . . . 604

Errata, . . . . . . . . . . . . . . 604

